# InN superconducting phase transition

**DOI:** 10.1038/s41598-019-48783-0

**Published:** 2019-08-23

**Authors:** Zhi-Yong Song, Liyan Shang, Zhigao Hu, JunHao Chu, Ping-Ping Chen, Akio Yamamoto, Ting-Ting Kang

**Affiliations:** 1 Key Laboratory of Polar Materials and Devices, Ministry of Education, East China Normal University, Shanghai, 200062 China; 2 State Key Laboratory of Infrared Physics, Shanghai Institute of Technical Physics, Chinese Academy of Sciences, 200083 Shanghai, People’s Republic of China; 3 University of Fukui, Fukui, 910-8507 Japan

**Keywords:** Electrical and electronic engineering, Superconducting properties and materials

## Abstract

InN superconductivity is very special among III–V semiconductors, as other III–V semiconductors (such as GaAs, GaN, InP, InAs, etc.) usually lack strong covalent bonding and thus seldom show superconductivity at low temperatures. Here, we probe the different superconducting phase transitions in InN highlighted by its microstructure. Those chemical-unstable phase-separated inclusions, such as metallic indium or In_2_O_3_, are intentionally removed by HCl acid etching. The quasi-two-dimensional vortex liquid-glass transition is observed in the sample with a large InN grain size. In contrast, the superconducting properties of InN with a small grain size are sensitive to acid etching, showing a transition into a nonzero resistance state when the temperature approaches zero. Since the value of ξ_0_ (the zero-temperature-limit superconducting coherence length) is close to the grain size, it is suggested that individual InN grains and intergrain coupling should be responsible for the sample-dependent InN superconducting phase transition. Our work establishes a guideline for engineering superconductivity in III-nitride.

## Introduction

Group-III nitrides are no doubt the typical semiconductors considered in optoelectronic applications^1–3^. Therefore, it is surprising that one of its members, InN (indium nitride), can be a superconductor at low temperatures^4,5^. Since the first report by Inushima *et al*.^6^, InN superconductivity and related physics are still not well resolved. It is known that a typical III–V semiconductor (e.g., GaAs, InAs, GaN, etc., except for BN, whose B and N atoms are bound by strong covalent bonds) is usually not easily made superconducting^7,8^. In contrast, IV semiconductors (e.g., Si, diamond, SiC, etc.) can become superconducting by heavy doping^9^. The reason is explained under the framework of the BCS mechanism: the IV semiconductor has strong directional covalent bonds, but the III–V semiconductor has weak covalent bonds. Therefore, the III–V semiconductor does not have a sufficiently large phonon energy and strong electron-phonon coupling. Additionally, the enhanced spin-orbit coupling in III–V tends to break the Copper pairing under the BCS mechanism. Along this line of thinking, GaN (which has a more covalent character) would more easily exhibit superconductivity than would InN. However, experimental evidence for GaN superconductivity is still lacking^7^. The traditional IV semiconductor superconductivity mechanism, which is driven by phonons strongly coupled to holes at the Γ point and requires the material to be p-type^10^, is not straightforwardly applicable in InN (the superconductivity of which is observed in n-type samples but not in p-type samples)^11–13^.

Although InN superconductivity seems to be “unreasonable” within the framework of current superconductivity theory for semiconductors, the major controversy against InN superconductivity does not stem from this point but from another simpler speculation: InN can have two phase-separated superconducting inclusions, i.e., indium metal (In) and indium oxide (In_2_O_3_), the superconductivity of which may “contaminate” InN. However, this naive In/In_2_O_3_ speculation faces strong challenges. Concerning In, its upper critical field H_c2_ (~0.03 T) is much smaller than that of InN (~1 T). Although a small In nanoparticle was theoretically proposed to increase H_c2_^14^, this proposal is not reliable because the small size effect has been experimentally proven to suppress the superconductivity in In^15^. Furthermore, another similar III-nitride, GaN, has been made superconducting by it containing a heavy amount of Ga but has a similar H_c_ to that of Ga^7,16^. Thus, it is less persuasive to assume that the metal nanoparticle superconductivity effect is present only in InN and not in other III-nitrides. Concerning In_2_O_3_, the attribution of the 33° XRD peak to In_2_O_3_ is not well recognized^17^, as metallic indium, amorphous InN, InN(10–11), and cubic InN can also produce similar XRD reflections^12^. Additionally, achieving In_2_O_3_ superconductivity is not easy and requires low disorder^18^. It is difficult to believe that the unintentionally introduced In_2_O_3_ in InN can meet such requirements.

In addition to its importance in superconductivity physics, InN superconductivity is of technical interest. For example, an InN superconductor can be used to fabricate a superconducting single-photon detector (SSPD) with the following justifications. First, compared with NbN, which is the most successful material in an SSPD^19,20^, InN shares many similar and attractive properties, namely, high chemical stability, good mechanical performance, etc. Second, InN has a suitable transition temperature T_c_ (~3 K). In contrast, high-T_c_ superconducting material is not suitable for SSPD applications, as its large superconducting gap energy will reduce the sensitivity to photons of a given energy (especially at longer wavelengths)^20^. On the other hand, too low T_c_ requires a large cooling power, so it is less convenient. Finally, the application of InN in the superconducting industry is favored because it can be integrated with the III–V semiconductor technology, allowing an easy combination of semiconductor and superconductor function within one material system, i.e., III-nitride.

In this work, we remove the In/In_2_O_3_ inclusions (which are highly reactive) in InN by acid etching and check the InN superconductivity. This experiment is not yet available in the literature. This work is also useful for future applications of InN in an SSPD, where chemical stability is preferred.

## Experimental Method

Two n-type InN samples (sample A and sample B) were grown by metalorganic vapor phase epitaxy (MOVPE) on insulative GaN/sapphire (0001) templates at the University of Fukui. A varying growth-temperature strategy^21^ is adopted to control the grain size of the InN crystal. The growth temperature is 520 °C (480 °C) for sample A (B). The detailed growth conditions and crystal structure characterizations can be found elsewhere^21^. The thickness of sample A (B) is measured to be ~850 nm (~950 nm) by cross-section SEM.

**Figure 1:**
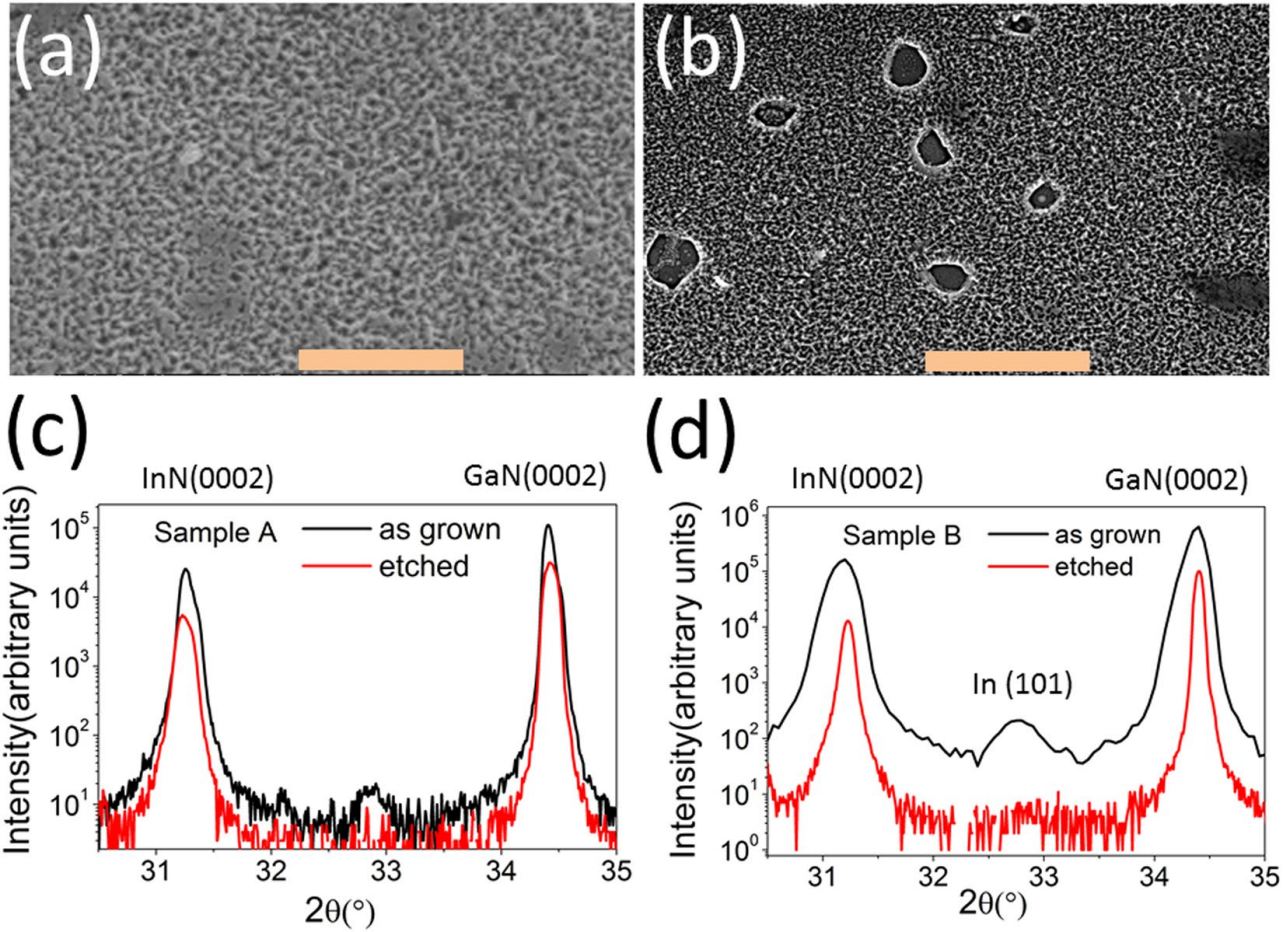
(**a,b**) SEM images for the etched samples A (**a**) and B (**b**); the scale bar length is 5 μm. (**c,d**) X-ray diffraction patterns of the as-grown, etched InN films of samples A (**c**) and B (**d**).

The room temperature electron mobility and concentration of sample A (B) is 505 cm^2^/Vs (280 cm^2^/Vs) and 1.2 × 10^19^ cm^−3^ (2.2 × 10^19^ cm^−3^). Figure 1(c,d) are the X-ray diffraction (XRD) results of samples A and B, respectively. In addition to the InN(0002) reflection at 31.36°, both samples have a small peak at ~33.0°, which can be removed by HCl acid etching. Therefore, it is attributed to In(101) (2θ = 32.95°)^22^ or (110) reflection of a rhombohedral phase In_2_O_3_ (2θ = 32.92°).

Each sample was cut into small pieces (with a size of ~1 mm × 10 mm) and subjected to HCl etching to remove the possible In/In_2_O_3_ inclusions. Figure 1(a,b) show the surface morphologies (SEM images) of the HCl etched samples. As seen in the figure, sample B shows more voids after HCl etching than does sample A, indicating more significant phase separation in sample B. Transport measurements were performed at the Shanghai Institute of Technical Physics with a dilution refrigerator down to a temperature of ~30 mK and a cryogen-free low-temperature system with 1.5 K as the lowest temperature. A magnetic field was applied vertical to the sample surface, i.e., along the InN c[0001] axis. The ohmic contacts were made from metal indium or silver pastes.

## Results and Discussions

### Demonstration of the chemical-stable InN superconductivity

**Figure 2:**
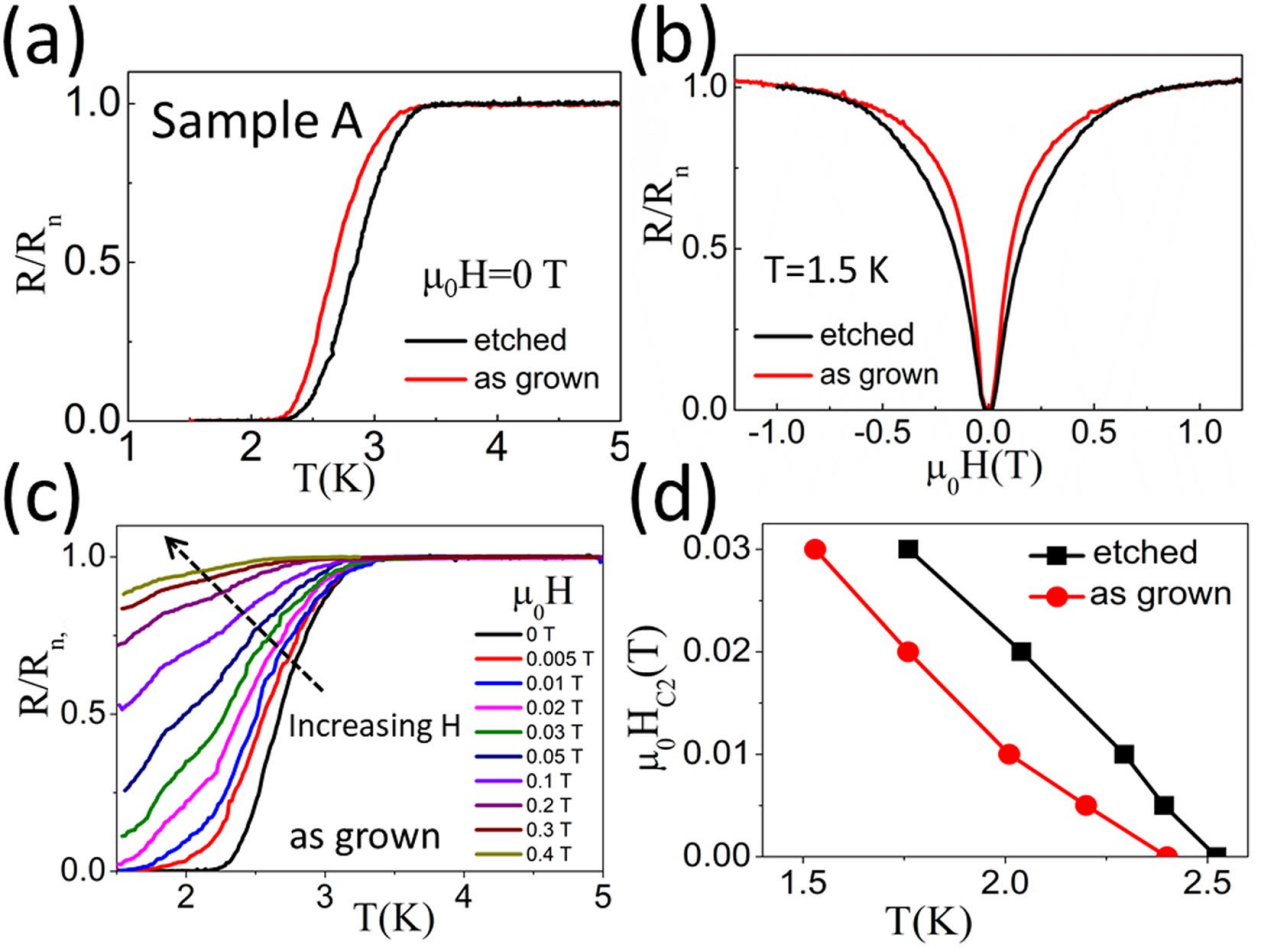
(**a**) R-T transitions and (**b**) R-H transitions for as-grown and etched sample A. (**c**) Normalized resistance as a function of temperature T under different magnetic fields H for as-grown sample A. (**d**) The upper critical field (H_c2_) as a function of temperature T for as-grown and etched sample A. In (**a**–**d**), the resistance is measured with a current of 1 μA.

Figure 2(a,b) display the normalized resistance R/R_n_ (R_n_ is the normal state resistance) as a function of temperature T (R-T) and magnetic field H (R-H) of sample A. Because the superconductivity is observed both in the as-grown and etched sample A, the superconductivity in InN is proven to be able to survive acid etching. The critical temperature and magnetic field are not changed significantly by etching. This indicates that the effect of those chemical unstable inclusions (e.g., large-sized In/In_2_O_3_) on InN superconductivity is very limited.

Figure 2(c) displays the R-T curves of the as-grown sample A under different magnetic fields (H). With increasing H, the superconducting transition broadens and shifts to lower temperatures. From these curves, we can deduce the upper critical field (H_c2_), which is defined as the H field when R/R_n_ = 10%. Figure 2(d) gives the deduced H_c2_ as a function of temperature T. From Fig. 2(d), we can calculate the dirty-limit coherence length (ξ_0,dirty_) of sample A by the dirty-limit (in the case of the mean free path *l* ≪ ξ_0_) relation^23^:

1a $${{\rm{H}}}_{{\rm{c}}2}(0)=0.69{{\rm{T}}}_{{\rm{c}}}\frac{{{\rm{dH}}}_{{\rm{c}}2}}{{\rm{dT}}}{|}_{{\rm{T}}={{\rm{T}}}_{c}}$$

1b $${{\rm{\xi }}}_{0,{\rm{dirty}}}={[{\varphi }_{0}/2{{\rm{\pi }}H}_{{\rm{c}}2}(0)]}^{1/2}$$

where mean-field T_c_(H) at different H is determined from R-T scans, in which R/R_n_ = 10% is met. Eq. (1) gives the ξ_0,dirty_ value for the as-grown/etched sample A as 76 nm/71 nm, respectively. The clean-limit (Pippard) coherence length ξ_0,clean_ of sample A is estimated by^24–26^:

2 $${{\rm{\xi }}}_{0,{\rm{clean}}}=\frac{{{\rm{V}}}_{{\rm{F}}}{\rm{\hslash }}\,}{{\rm{\pi }}{\rm{\Delta }}}=\frac{0.1804{{\rm{V}}}_{{\rm{F}}}{\rm{\hslash }}}{{{\rm{k}}}_{{\rm{B}}}{{\rm{T}}}_{{\rm{C}}}}$$

where 2Δ = E_g_ is the superconducting energy gap, V_F_ is the fermi velocity, and T_c_ = 2.4 K is the transition temperature where R/R_n_ = 10% occurs. It reaches ξ_0,clean_ ≈ 710 nm.

In a conventional superconductor, the coherence length ξ(T) will approach ξ_0_ in the limit of T → 0. Meanwhile, near T_c_, ξ(T) diverges as (T_c_-T)^−1/2^. ξ_0_ is equal to ξ_0,clean_ if the sample is “clean” and to ξ_0,dirty_ if it is “dirty” (i.e., when the mean free path *l* ≪ ξ_0_)^26^. By the Hall measurements and InN effective electron mass *m*_e_*/*m*_0_ = 0.07^5^, we reach *l* ≈ 25 nm for sample A. Therefore, the dirty limit requirement *l* ≪ ξ_0,dirty_ is not met well^23^, and ξ_0_ will be close to ξ_0,clean_. Considering the fact that *l* < ξ_0,dirty_ < ξ_0_ < ξ_0,clean_ here, ξ_0_ is believed to lie in the range of 100–1000 nm.

Note that this InN ξ_0_ range (i.e., 100–1000 nm) deduced above is consistent with the previous magnetic susceptibility measurements on the MOVPE InN sample (also from Fukui and with *l* ~ 38 nm) in ref.^12^. From ref.^12^, where the “H_c2_” (~4 mT) and “T_c_^”^(~1.7 K) values are reached by the magnetic susceptibility results, the following results hold: ξ_0,dirty_ ~ 290 nm and ξ_0,clean_ ~ 820 nm, which sets the ξ_0_ range to 290 nm–820 nm in ref.^12^. Therefore, the InN ξ_0_ values measured by different methods are not far from each other and lie mainly in the range of 100–1000 nm.

Previously, the largest grain size of our MOVPE InN was observed to be ~500 nm^21^, which agrees with the morphology in Fig. 1(a). Therefore, ξ_0_ is now close to the grain size^21^ of MOVPE InN and will make difference in the superconducting properties of InN. Since ξ_0_ is the characteristic size that permits the occurrence of superconductivity^15,25^, an InN sample with a smaller grain size will be more sensitive to microstructure details with respect to superconductivity, which we will discuss later.

### The basic features of the InN superconducting phase transition: quasi-two-dimensional vortex liquid-glass transition

Although zero resistance is the major characteristic of a superconductor, an understanding of the superconductor’s vortex dynamics (which causes dissipation and thus resistivity) and the resulting superconducting phase transition is necessary. Among various experimental methods, the current-voltage (I–V) measurements at different temperatures and magnetic fields can provide important information on the phase transition. On the other hand, the nonlinear and scaled I–V curves (see below) can further support the idea that InN is truly a superconductor rather than a normal metal with very low resistivity.

**Figure 3:**
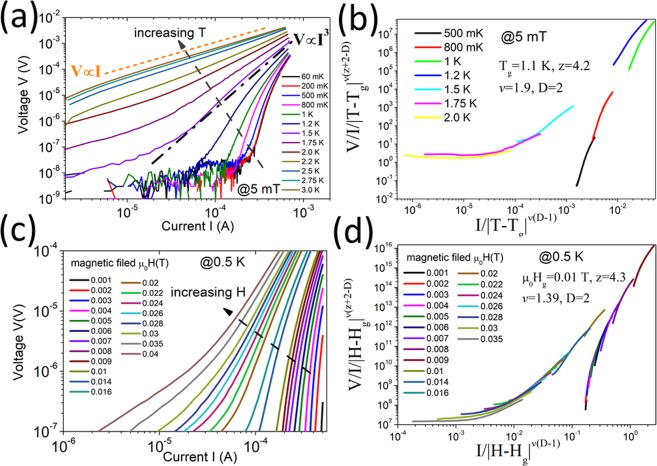
For etched sample A, (**a**) I–V curves at various temperatures from 60 mK to 3 K for μ_0_H = 5 mT. (**b**) VG scaling of the I–V curves in (**a**). (**c**) I–V characteristics measured at 0.5 K for magnetic fields ranging from 0 to 40 mT. (**d**) VG scaling of the I–V curves in (c**)**.

For the etched sample A, Fig. 3(a) shows the I–V curves under different temperatures T, with a small magnetic field μ_0_H = 5 mT (for introducing the vortex). Figure 3(c) displays the I–V curves under different magnetic fields H, with a temperature of 0.5 K (≪T_c_). The I–V curves show a smooth evolution in the curvature from convex to linear with increasing T and H. These phenomena agree well with the vortex-glass (VG) theory, where the vortex comes from the self-field due to a persistent current and the applied magnetic field is pinned at small T/H (which results in the “convex” I–V) and then depinned at large T/H (which results in the linear I–V).

In VG theory, with the decrease in T, the mobile vortex (i.e., vortex-liquid state) will finally be pinned by the randomly distributed pinning centers, reaching the superconducting state (i.e., vortex-glass state). Therefore, the I–V curves at different temperatures near the liquid-glass phase transition temperature (T_g_) can be scaled into two different branches by the scaling law^27–29^:

3 $$\frac{{\rm{V}}}{{\rm{I}}{({\rm{T}}-{{\rm{T}}}_{{\rm{g}}})}^{{\rm{\nu }}({\rm{z}}+2-{\rm{D}})}}={{\rm{f}}}_{\pm }(\frac{{\rm{I}}}{|{\rm{T}}-{{\rm{T}}}_{{\rm{g}}}{|}^{{\rm{\nu }}({\rm{D}}-1)}})\,$$

where *v* is the exponent of the vortex-glass correlation length ξ_g_ and ξ_g_ diverges at T_g_ as:

4 $${{\rm{\xi }}}_{{\rm{g}}} \sim {|{\rm{T}}-{{\rm{T}}}_{{\rm{g}}}|}^{v}$$

z is the dynamical critical exponent, D is the number of dimensions, and f_±_ are the scaling functions above and below T_g_. Above T_g_, being in the vortex-liquid phase, there is a finite linear resistance for a low current limit I → 0^27^:

5 $${{\rm{R}}}_{{\rm{lin}}}={({\rm{V}}/{\rm{I}})}_{{\rm{I}}\to 0}\propto {({\rm{T}}-{{\rm{T}}}_{{\rm{g}}})}^{{\rm{\nu }}({\rm{z}}+2-{\rm{D}})}$$

and the I–V curve becomes nonlinear for large I. At T_g_, the I–V curve satisfies the relationship:

6 $${\rm{V}}({\rm{I}}){|}_{{\rm{T}}={{\rm{T}}}_{{\rm{g}}}} \sim {{\rm{I}}}^{{\rm{z}}+1/{\rm{D}}-1}$$

Below T_g_, being in the vortex-glass regime, the double-logarithmic I–V curves have downward curvatures corresponding to a vanishing linear resistance. The scaling results are shown in Fig. 3(b). The I–V curves are nicely scaled into two branches, which touch each other at the T = 1 K−1.2 K scaling curves. This agrees well with the fitting T_g_ value T_g_ = 1.1 K.

Figure 3(a) can be fully explained within the framework of VG theory. When temperature T < T_g_, the sample is in the vortex-glass state. Thus, the resistance falls rapidly with decreasing current and is zero below the critical current. At T > T_g_, the sample is in the vortex-liquid state, the I–V curves exhibit ohmic behavior and the resistance remains constant even at small current I. Previous theoretical and experimental studies show that z = 4~7 and ν = 1~2 are reasonable values for the VG phase transition^27^. To obtain a good scaling performance, we have D = 2, ν = 1.9, and z = 4.2.

Since the magnetic field and temperature exhibit analogous effects in suppressing superconductivity and generating quasi-particles in conventional superconductors, the I–V relations under different magnetic fields, which are shown in Fig. 3(c), are qualitatively similar to the I–V curves at different temperatures in Fig. 3(a). After scaling, these I–V curves are separated into two branches by a particular magnetic field (μ_0_H_g_). We can modify Eq. (3) by replacing T_g_ with H_g_, based on VG theory^27^. Then, those IV curves can also be scaled into two branches, with μ_0_H_g_ = 10 mT and D = 2.

D = 2 means that the VG transition in Fig. 3 is quasi-two-dimensional (quasi-2D)^29,30^. This is further supported by two behaviors in I–V. First, as shown in Fig. 3(a), for temperatures in the range 1.5 K-2 K (i.e. the vortex-liquid region), the I–V curves exhibit a crossover from linear behavior at small current I to nonlinear behavior at high I. This crossover is anticipated in the quasi-2D VG model: at low I, the vortex dynamics at length scales larger than ξ_g_ (ξ_g_ is on the order of ξ) is considered, where the system will behave like a vortex liquid with a linear resistance; at high I, the excitations involve length scales smaller than ξ_g,_ and the associated glassy dynamics yields a nonlinear I–V relation^29^. Second, a Berezinskii-Kosterlitz-Thouless (BKT) transition^30–32^, which is a characteristic phase transition in a 2D superconductor, was found at a specific temperature T_BKT_ = 1.5 K in Fig. 3(a) by the occurrence of V ∝ I^3^ ^30^. Below T_BKT_, the vortex/antivortex pairs generated by topological excitations will be bound, leading to a zero linear resistivity. At T_BKT_, V ∝ I^3^, which is a sign of the BKT transition. The observed VG transition agrees with previous reports that InN is a type-II superconductor^12^. The quasi-2D VG character indicates that the thickness of sample A, i.e., ~900 nm, is comparable or smaller than the ξ_0_ and the corresponding ξ_g_.

### The modified InN superconducting phase transition

**Figure 4:**
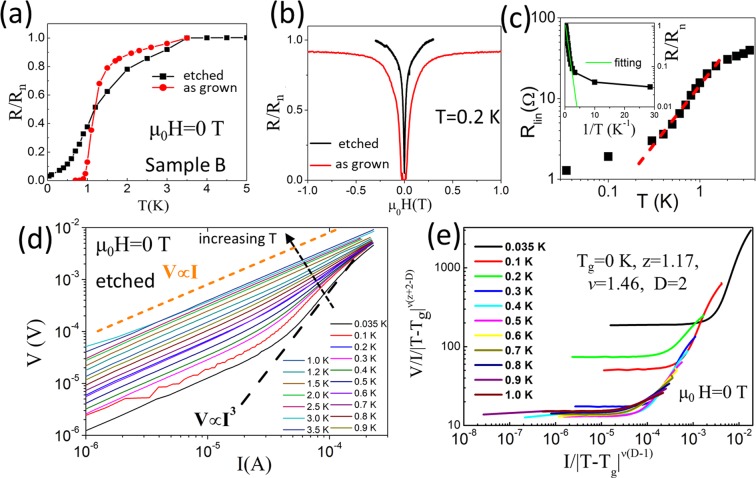
(**a**) R-T transitions and (**b**) R-H transitions for as-grown and etched sample B. (**c**) The linear resistance R_lin_ at I → 0 [deduced from the I–V curves in Fig. 4(d)] as a function of temperature for etched sample B. (**d**) I–V curves measured at magnetic field μ_0_H = 0 T for etched sample B. (**e**) Scaling of the I–V curves in (**d**).

Different from sample A, sample B is sensitive to acid etching. Figure 4(a) displays the R-T curves of the etched sample B. Compared with the as-grown sample B, the superconducting transition is greatly broadened after etching. In addition, the resistance of the etched sample B is not zero even at the lowest temperature T ≈ 35 mK. The R-H measurements in Fig. 4(b) show that the “critical” magnetic field of etched sample B is also significantly reduced.

Figure 4(d) shows the I–V curves of etched sample B at different temperatures. With increased temperature, the I–V curves show an evolution from convex curvature to linear dependence, which indicates that the superconducting vortex-glass state does not exist even at the lowest temperature, i.e., ~35 mK. The trend toward the BKT transition with V ∝ I^3^ is observed under reduced temperature. The resistance goes to a finite value as the current I approaches zero, i.e., V ∝ I at I → 0. In Fig. 4(e), we try to scale the I–V curves, but the attempt fails, and the two-branch characteristic in the VG transition cannot be produced. The fitting parameters also cannot be adjusted to the reasonable range, e.g., z = 1.17 is outside the z = 4~7 range.

The failed scaling is mainly due to the appearance of a nonzero resistance state (NZRS) at the limit of T → 0^33^. As shown in Fig. 4(c), the NZRS is not a vortex-liquid state with T_g_ = 0 K, as the deduced I → 0 linear resistance R_lin_ is not linear to log(T), as required by Eq. (5)^30^. This NZRS is also inconsistent with other mechanisms that can suppress the superconductivity, such as phase slip (PS), in which the phase of the superconducting condensate wave-function jumps irreversibly, leading to the nonzero resistance. These phase slips represent activation processes that can be triggered either thermally (i.e., thermally activated phase slip, TAPS) or through quantum tunneling (quantum phase slip, QPS)^33^. However, a TAPS requires a temperature dependence of the resistance as^33^:

7 $${\rm{R}}({\rm{T}})\propto \exp (-{\rm{U}}/{{\rm{k}}}_{{\rm{B}}}{\rm{T}})$$

where U is the activation energy. However, NZRS does not follow Eq. (7) at T → 0, as shown in Fig. 4(c, inset). On the other hand, the QPS explanation requires that the sample sheet resistance R_s_ in the normal state is larger than the quantum resistance R_Q_ = h/4e^2^ = 6.45 kΩ ^34^, while the R_s_ of our InN sample is too small (~10 Ω).

According to the phase transition theory, the scaling behavior is attributed to the existence of a characteristic length that diverges at the transition temperature^10^, as in Eq. (4). Therefore, we propose that there should be a special size that limits the expansion of the vortex-glass correlation length ξ_g_ in the etched sample B even at T → 0 K. We believe that the InN grain size can be such a special size, where the intergrain boundary can destroy the correlation among the vortex in nearby grains. On the other hand, since NZRS is not observed in the as-grown sample B, as shown in Fig. 4(a), the In/In_2_O_3_ inclusion seems to “suppress” the NZRS.

**Figure 5:**
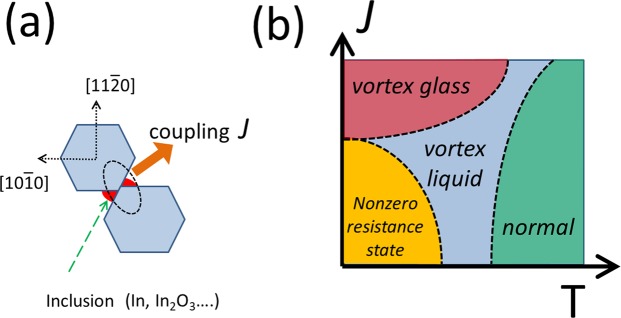
(**a**) Schematics of the intergrain superconducting coupling in InN(0001) film. The presence of intergrain inclusion can enhance the intergrain coupling J. If these inclusions are removed, the grain boundary can serve only as a weak link of small J. (**b**) Schematic phase diagram of InN superconductor with the presence of a vortex (under a small magnetic field or with the persistent current’s self-field) as a function of J and temperature T.

Figure 5(a) gives our theoretical picture regarding the NZRS. In this picture, we suggest that two factors in the microstructures are highlighted in InN superconductivity. First, the grain size L makes a difference. If L is comparable to or smaller than the coherence length ξ_0_ (e.g., ~700 nm or less), the magnitude fluctuations of the superconducting order parameter will destroy the superconductivity^15^. Consequently, this grain will no longer be superconducting. Second, the intergrain coupling can recover the superconductivity in InN. In this case, the diffusion of electron pairs from the superconductor grain into the normal material (known as the proximity effect) and then into the neighboring superconductor grain gives rise to global superconductivity.

Figure 5(b) gives the schematic phase diagram describing InN superconductivity with the presence of vortex as a function of intergrain coupling J and temperature T. Since superconductivity in an individual grain is fragile owing to its small size L < ξ_0_ or L ~ ξ_0_, nonsuperconducting T = 0 states are presented as the NZRS. The appearance of the NZRS signifies the existence of a mesoscopic energy scale on each grain (e.g., an effective “charging energy” or the electronic level spacing on a grain, etc.)^15,32^. Therefore, a minimum interisland coupling J_m_ must be overcome to obtain the global superconductivity^15^.

### Discussion on the sample-dependent superconducting phase transition in InN

With the phase diagram in Fig. 5(b), which is based on the grain size and intergrain coupling J, we can consistently understand the various sample-dependent superconductivity results for InN. For those small grain size InN films (e.g., sample B), since the superconductivity in each grain is fragile or even not present, the sample’s global superconductivity heavily relies on the intergrain coupling, which is realized by the intergrain inclusion In/In_2_O_3_. Previous reports^22^ also clearly demonstrated that indium metal prefers to reside on the (11–20) planes of InN, which is the intergrain interface of InN. Acid etching can remove such inclusions and reduce the intergrain coupling J. Consequently, a broadened superconducting transition does result not only simply from a spread in T_c_ of the individual grains but also from an inhomogeneous distribution of J. The NZRS will then appear due to J < J_m_. For an InN film with a large grain size (e.g., sample A), its individual grains have a robust superconductivity with a rather uniform T_c,_ and its intergrain coupling is already strong (because of the large area of the intergrain directly touching the interface without the presence of In/In_2_O_3_ inclusions). Therefore, acid etching has no obvious influence. The morphology observed in Fig. 1(c,d) and in previous reports^21^ agrees with the grain-size attribution above.

On the roles of In/In_2_O_3_ in InN superconductivity, we believe that they can enhance the superconductivity by filling the gaps between InN grains. However, if In or In_2_O_3_ alone wants to establish global superconductivity, it must (a) continuously distribute from the source to the drain and undergo exposure there and (b) have a grain size > ~30 nm for an In/In-oxide composite film^35^ or >4 nm for pure metal In^15^. However, items (a) and (b) will make In/In_2_O_3_ easy to etch away by acid, thus removing the superconductivity. Kadir *et al*. claimed that In_2_O_3_ alone contributes to the superconductivity in InN via a thermal annealing experiment^17^. However, their annealing temperature was too high (530–650 °C) compared with the growth temperature. The grain size and intergrain coupling were certainly modified there.

## Summary

We prove that InN superconductivity can be chemically stable, which supports InN as an intrinsic superconductor. This paves the way for the future field of semiconductor-superconductor integrated electronics (or optoelectronics) within only one material family (i.e., III-nitride). For example, the InN superconducting single-photon detector (SSPD) can be equipped with an on-wafer amplifying circuit made by a GaN/AlGaN HEMT (high electron mobility transistor). InN is obviously more suitable for semiconductor-superconductor integration than the previously suggested NbN superconductor^36^. On the other hand, we show that the InN superconductivity property can be a function of its grain size and the intergrain inclusions. If the intergrain inclusions play an important role in coupling the superconductivity between nearby InN grains, their removal can greatly modify the superconducting behaviors of InN. This provides a novel method for controlling the superconductivity properties of III-nitride by engineering the crystal’s microstructures.

## References

[1] Akasaki I (2015). Nobel Lecture: Fascinated journeys into blue light. Rev. Mod. Phys..

[2] Amano H (2015). Nobel Lecture: Growth of GaN on sapphire via low-temperature deposited buffer layer and realization of p-type GaN by Mg doping followed by low-energy electron beam irradiation. Rev. Mod. Phys..

[3] Nakamura S (2015). Nobel Lecture: Background story of the invention of efficient blue InGaN light emitting diodes. Rev. Mod. Phys..

[4] Bhuiyan AG, Hashimoto A, Yamamoto A (2003). Indium nitride (InN): A review on growth, characterization, and properties. J. Appl. Phys..

[5] Wu J (2009). When group-III nitrides go infrared: New properties and perspectives. J. Appl. Phys..

[6] Inushima T (2001). Physical properties of InN with the band gap energy of 1.1 eV. J. Cryst. Growth.

[7] Iakoubovskii K (2009). Recent advances in superconductivity of covalent superconductors. Physica C.

[8] Bustarret E (2015). Superconductivity in doped semiconductors. Physica C..

[9] Blase X, Bustarret E, Chapelier C, Klein T, Marcenat C (2009). Superconducting group-IV semiconductors. Nature material.

[10] Bustarret E (2006). Superconductivity in doped cubic silicon. Nature.

[11] Xie W (2017). Transport properties for Zn+ ion implanted InN films at low temperature. Materials Letters.

[12] Inushima T (2012). Superconducting Properties of InN with Low Carrier Density near the Mott Transition. J. Phys. Soc. Jpn..

[13] Tiras E, Gunes M, Balkan N, Airey R, Schaff WJ (2009). Superconductivity in heavily compensated Mg-doped InN. Appl. Phys. Lett..

[14] Komissarova TA, Parfeniev RV, Ivanov SV (2009). Comment on “Superconductivity in heavily compensated Mg-doped InN”. Appl.Phys. Lett..

[15] Jaeger HM, Haviland DB, Orr BG, Goldman AM (1989). Onset of superconductivity in ultrathin granular metal films. Phys. Rev. B.

[16] Alekseevskii NE, Samsonov GV, Shulishova OI (1963). Superconductivity of Gallium Nitride. Sov. Phys. JETP.

[17] Kadir A (2008). Non-intrinsic superconductivity in InN epilayers: Role of Indium Oxide. Solid State Commun..

[18] Shahar D, Ovadyahu Z (1992). Superconductivity near the mobility edge. Phys. Rev. B.

[19] Hadfield RH (2009). Single-photon detectors for optical quantum information applications. Nature Photonics.

[20] Natarajan CM, Tanner MG, Hadfield RH (2012). Superconducting nanowire single-photon detectors: physics and applications. Supercond. Sci. Technol..

[21] Yamamoto A, Kodama K, Shigekawa N, Matsuoka T, Kuzuhara M (2016). Low-temperature (≥400 °C) growth of InN by metalorganic vapor phase epitaxy using an NH3 decomposition catalyst. Jpn. J. Appl. Phys..

[22] Kang T-T (2006). InN nanoflowers grown by metal organic chemical vapor deposition. Appl. Phys. Lett..

[23] Chockalingam SP (2008). Superconducting properties and Hall effect of epitaxial NbN thin films. Phys. Rev. B.

[24] Poole, C. P., Jr., Farach, H. A., Creswick, R. J. & Prozorov, R., *Superconductivity*, 2nd ed. P149 (Academic Press, 2007).

[25] Bezryadin, A. *Superconductivity in Nanowires: Fabrication and Quantum* Transport, 2nd ed (Wiley-VCH, Weinheim, 2013).

[26] Tinkham, M. *Introduction to superconductivity*, 2nd ed. (McGraw-Hill, NewYork, 1996).

[27] Sun Y (2013). Voltage-current properties of superconducting amorphous tungsten nanostrips. Sci. Rep..

[28] Huse DA, Fisher MPA, Fisher DS (1992). Are superconductors really superconducting?. Nature.

[29] Dekker C, Wöltgens PJM, Koch RH, Hussey BW, Gupta A (1992). Absence of a finite-temperature vortex-glass phase transition in two-dimensional YBa_2_Cu_3_O_7−δ_ films. Phys. Rev. Lett..

[30] Yang H (2007). I–V characteristics of the vortex state in MgB2 thin films. Phys. Rev. B.

[31] Resnick DJ, Garland JC, Boyd JT, Shoemaker S, Newrock RS (1981). Kosterlitz-Thouless Transition in Proximity-Coupled Superconducting Arrays. Phys. Rev. Lett..

[32] Eley S, Gopalakrishnan S, Goldbart PM, Mason N (2012). Approaching zero-temperature metallic states in mesoscopic superconductor–normal–superconductor arrays. Nature Phys.

[33] Baumans XDA (2016). Thermal and quantum depletion of superconductivity in narrow junctions created by controlled electromigration. Nat. Commun..

[34] Fisher MPA (1986). Quantum Phase Slips and Superconductivity in Granular Films. Phys. Rev. Lett..

[35] Fiory AT, Hebard AF, Glaberson WI (1983). Superconducting phase transitions in indium/indium-oxide thin-film composites. Phys. Rev. B.

[36] Yan R (2018). GaN/NbN epitaxial semiconductor/superconductor heterostructures. Nature.

